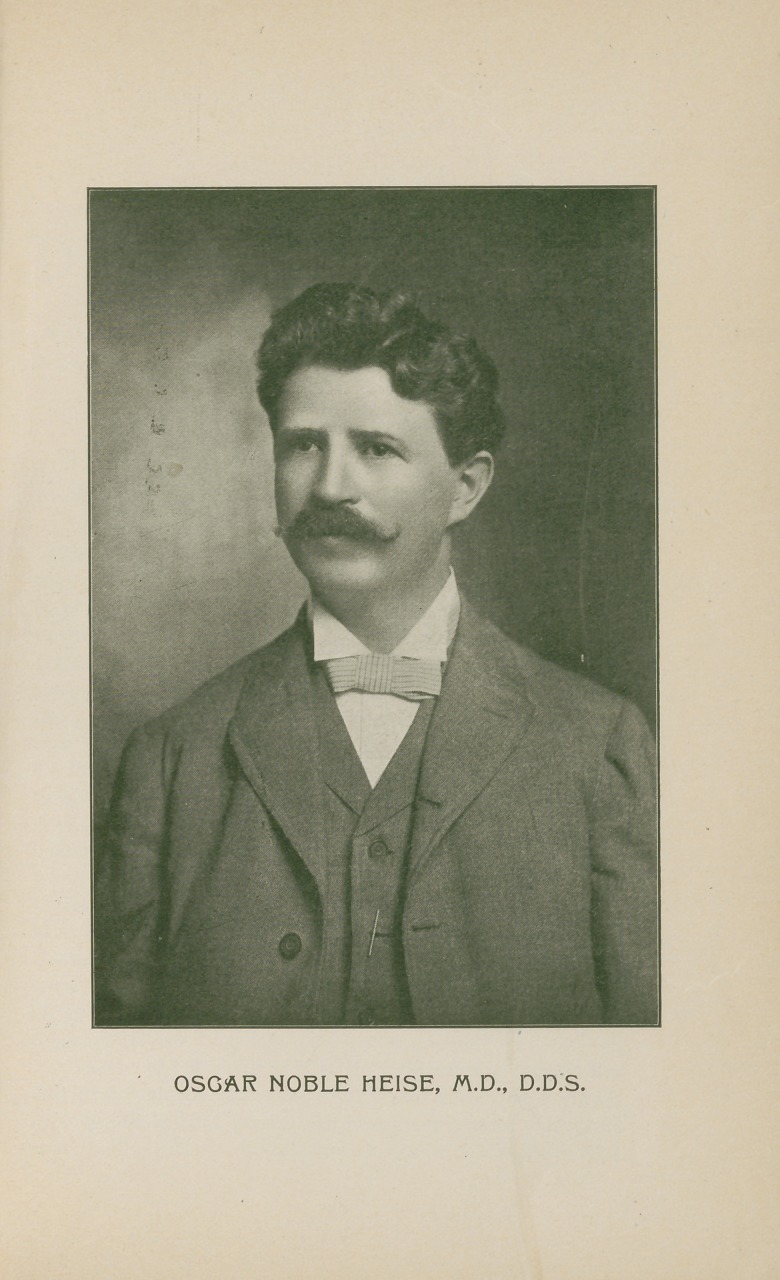# Obituary

**Published:** 1906-06-15

**Authors:** 


### OBITUARY.

---

Dr. Oscar Noble Heise, of Cincinnati, died May 15th, 1906, in the forty-sixth year of his age, of carcinoma of the intestines.

It will be sad news to many in the profession to learn of the death of Dr. Heise, as he was well known throughout the country, but it was not generally known that he has been fighting this disease for over three years, only his intimate friends have known it, and they were prepared for the fatal termination.

Dr. Heise was born at New Bremen, Ohio, October 18, 1860, where his father, Rev. Carl Heise, was pastor of the German Lutheran Church. The father was a fine scholar, being educated at the University of Halle, where he lived. He began teaching in the families of the nobility and studied theology. He preached in the German Lutheran Church of Halle for five years before coming to America. Naturally he took charge of the instruction of his own children, and Oscar did not enter the public schools until he was twelve years of age and well advanced in his primary education.

In 1877 he came to Cincinnati to visit relations and concluded to take up the study of dentistry, and because the students of Dr. E. G. Betty, who had his office in the same building with the editor of the *Register*. Consequently we saw much of Dr. Heise at the beginning of his professional career, and learned very soon to admire those qualities of mind and heart which in later years won for him distinction as a scientific student and fame as a professional man and successful practitioner of the art of dentistry. Dr. Heise began practice in Cincinnati immediately after graduating from the Ohio College of Dental Surgery in 1879, and succeeded quickly in establishing himself in a lucrative practice. Not being satisfied with his medical scientific attainments, he took time from his practice to work out the medical degree in the Ohio Medical College from which he graduated 1886. He was a most indefatigable student, and became so much interested in the study of Medical science that he decided to add some specialty of medical practice to his dental practice. In 1890 he spent several months in a poly-clinic of New York City, making a special study of nose and throat diseases, which he then added to his practice of dentistry. In 1893 he spent the summer in German and Austrian Universities, perfecting his knowledge of the treatment of nose and throat diseases, in which he became very much interested, and afterwards practiced them with great success. Although he did a considerable amount of this special practice, he never allowed it to encroach upon his dental practice which he loved more than even his medical studies.

He was a constant attendant and worker in the local, state and national dental societies. Although he did not write as much as some others, nor engage in the debates of society meetings to any considerable extent—due largely to his modest nature—yet when he did write or speak, he always received the closest attention, as he spoke with the authority of a shcolar. He was a member of the American Medical Association, and took great interest in the work of the section on stomatology. He believed that dentistry was truly a specialty of medicine and longed for the time when it should be so accepted. He was an active supporter of his state society and served it most efficiently in various official capacities. For several years he was a member of the Ohio State Board of Dental Examiners and did excellent work there in elevating its standards of examinations and in dignifying the object for which the Board was organized. In his home city he was active in the conduct of the Cincinnati Odontological Society, which he helped to organize, and all the members of this society will recall with profit and pleasure the ability and generosity with which he gave of his knowledge and skill to its proceedings. It is a distinct loss to the profession to loose from its activities such an able and willing votary, and our sympathy is most cordially extended to the Ohio Societies which have benefited so much because of his loyal devotion to their interests. A large number of patients, friends and acquaintances in his home city will miss his ministration and cordial greetings, and his intimate professional friends will experience the loss of not only an able counsellor, but a true friend.

In June 1884, Dr. Heise was married to Miss Amelia L. Marqua, who with a daughter survives him. The loss of so gentle and loving a father must come as a severe blow to them, and the most cordial sympathy of all who knew Dr. Heise will be most fully extended to them in their great bereavement. We reproduce an excellent likeness of Dr. Heise in another part of this journal.

## Figures and Tables

**Figure f1:**